# ENVIRONMENTAL EXPOSURE-RELATED HEALTH WORRIES, WORK ABILITY AND HEALTH, SURVEYED BY OCCUPATIONAL HEALTH SERVICES

**DOI:** 10.13075/ijomeh.1896.02626

**Published:** 2025

**Authors:** Minna Majuri, Aki Vuokko, Mikko Korhonen, Kirsi Karvala, Markku Sainio

**Affiliations:** 1 University of Helsinki, Faculty of Medicine, Department of Public Health, Helsinki, Finland; 2 HUS Helsinki University Hospital, Occupational Health Services, Helsinki, Finland; 3 Finnish Institute of Occupational Health, Department of Occupational Medicine, Helsinki, Finland; 4 Tampere University, Faculty of Information Technology and Communication Sciences, Tampere, Finland; 5 Varma, Helsinki, Finland; 6 HUS Helsinki University Hospital, Rehabilitation Unit for Persistent Symptoms, Helsinki, Finland

**Keywords:** risk perception, work ability, occupational health services, environmental exposures, functional impairment, health worries

## Abstract

**Objectives:**

Environmental intolerance (EI) can negatively impact well-being and daily life, and even lead to disability. Healthcare can detect EI early and conduct interventions. This study explored ways of identifying environmental exposure-related health worries and EI during occupational health (OH) check-ups, and their associations with unselected working-age employees' perceived work ability, stress and overall health.

**Material and Methods:**

A crosssectional survey was conducted among 355 employees attending OH check-ups at an occupational health services (OHS) unit in Southern Ostrobothnia, Finland. Health worries about environmental exposures were measured using 2 single-item questions, one on exposures in general, the other on indoor air. Cutoffs were set for excessive worries. Environmental intolerance was defined using the *Quick Environmental Exposure and Sensitivity Inventory* (QEESI). Perceived stress, work ability and health were inquired. The analyses used descriptive statistics, Fisher's exact test and linear regression.

**Results:**

Participants with EI (N = 25, 7%) reported significantly poorer work ability and health, and higher stress than those without EI. Environmental intolerance was also associated with comorbid diseases such as asthma, migraine, mental disorders and irritable bowel syndrome. Those with excessive health worries about environmental exposure (N = 73, 21%) and indoor air (N = 182, 51%) outnumbered and mostly included those with EI. All the participants' (N = 355) increased health worry about environmental exposures was independently associated with poorer work ability and health, and higher perceived stress. The health worry questions for identifying EI were sensitively phrased, and the general question demonstrated good specificity.

**Conclusions:**

The findings show that environmental exposure-related health worries can be detected by and EI identified by single questions. Their interrelation and association with poorer work ability and health suggest they are part of the same continuum of increasing environmental worries and exposure-related reactions. Identifying health worries enables early detection and interventions such as psychoeducation, to prevent any related disability and adverse health outcomes.

## Highlights

- Environmental-related health worries are associated with poorer work ability.
- Environmental worries can be detected by single question.
- Identifying health worries enables early detection and interventions.

## INTRODUCTION

Environmental intolerance (EI) is characterized by recurring, nonspecific symptoms in multiple organ systems, which are attributed to minimal or non-existent exposure to various environmental factors [1,2]. The severity of EI can range from mild annoyance to significant disability, and fear-avoidance behaviour reactions related to perceived harmful exposures [1]. Although EI is frequently linked to chemicals [1,2], certain buildings [3] and electromagnetic fields [1], it may also relate to any environmental factor with a nocebo label [1,2]. The more severe the EI related to disability, the more co-occurrences of comorbidities, psychological distress and various EIs are observed [4–6].

Instead of medical or toxicological mechanisms [1,7], research has revealed that EI has a biopsychosocial nature, emphasizing the role of symptom perception, awareness and interpretation [1,2].

Individuals with EI display not only excessive reactivity but also heightened worry about the possible adverse health effects of environmental factors [8]. Double-blind provocation studies using cognitive cues have shown that symptoms and reactions can be influenced and initiated by nocebo expectations [1]. This highlights the involvement of central nervous system mechanisms, i.e., selective attention to and heightened perception of bodily sensations, catastrophizing interpretations, dysfunctional health behaviours, and somatosensory amplification [1,9]. Perceiving harmful environmental factors can activate the central stress axis, engaging the autonomic, immune, and endocrine systems, which underlie symptoms and reactions [1,10]. The interplay between interpretations that environments pose health risks and symptom attribution to environmental exposure can create a vicious circle of adverse personal, occupational and social consequences [10,11]. However, existing definitions and identification procedures of EI [1,2] including screening tools such as the *Quick Environmental Exposure and Sensitivity Inventory* (QEESI) [12], centre on symptoms and environmental exposures and overlook the role of related risk perceptions.

The link between environmental exposure-related health worries and EI with symptoms, illness behaviour and disabilities [1,13] suggests that the assessment of environmental risk perception should be incorporated into health screenings so that any adverse factors affecting health and work ability could be identified. Symptom attribution and exposure-related health worry play a substantial role in developing and maintaining ill health in individuals with EI [2,14,15]. Stress, fear and negative perceptions contribute to disability [10,14] and can serve as targets for effective interventions [9,10,16]. Therefore, enhancing early detection of and intervention in EI and excessive risk perceptions could be a new target for disability prevention. This study aims to investigate the identification of environmental exposure-related health worries and EI and their associations with work ability, perceived stress levels and health in a sample of unselected working-age employees attending health check-ups in occupational health services (OHS).

## MATERIAL AND METHODS

### Study population

This cross-sectional questionnaire study examined participants recruited from among employees of the City of Seinäjoki in Southern Ostrobothnia in Finland, who attended preventive occupational health (OH) check-ups in 2016–2018.

In Finland, OHS units serve as the primary healthcare providers for employees, covering 95% of the workforce. A key focus of these units is prevention, as they conduct mandatory preventive health check-ups based on workplace risk assessments, and aim to monitor employee health and prevent work-related illnesses or risks. The OHS units also offer voluntary health check-ups as part of health surveillance, which include elements of work ability evaluation and support, further emphasizing their preventive role. They may also provide care for non-work-related illnesses.

The survey was offered to employees during their statutory and voluntary health check-ups at their OHS units. The authors excluded those who had contacted OHS due to illness or work-related symptoms. The participants were recruited by 3 receptionists during the health check-up registration process and by ten OH nurses during the health check-ups. They received both oral and written information about the study and provided their signed informed consent to participate. They completed the questionnaire on an iPad during the OHS visit, via an email link after the check-up, or on paper.

**Figure 1:**
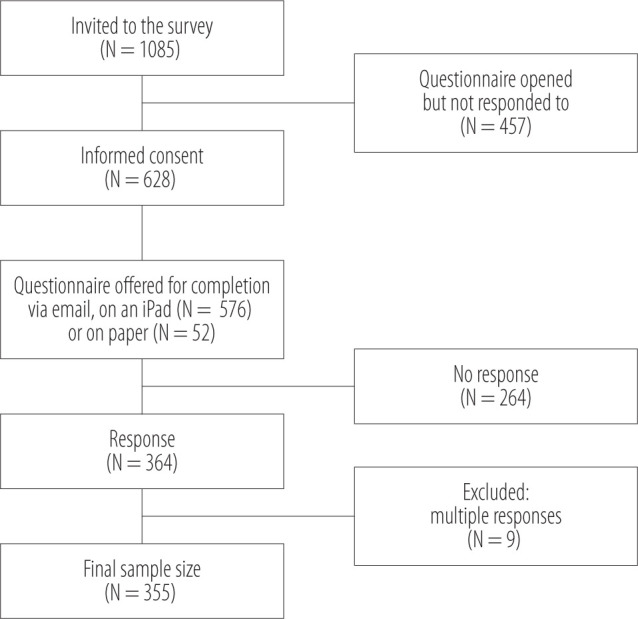
Flowchart of survey conducted during health check-ups in occupational health services (OHS) units in 2016–2018, Seinäjoki, Finland

During data collection, 4610 employees underwent health check-ups. Of these, 23.5% (N = 1085) were offered the study questionnaire, of whom 57.9% (N = 628) signed their written consent. Although 3 reminder emails were sent out, 264 participants who had consented did not complete the questionnaire. The final dataset consisted of the 355 employees (32.7%) who had completed the questionnaire (Figure 1).

### Ethics

The study was approved by the Tampere University Research Ethics Board (ETL code R14137), and was conducted in accordance with the Declaration of Helsinki. Data privacy was strictly followed.

### Questionnaire

The questionnaire assessed the presence and degree of health worries about environmental exposures, EI, perceived stress, current health and work ability. The authors also collected information on demographics such as age, gender, occupational group and physician-diagnosed diseases.

The degree of health worries about environmental exposures was evaluated using a single-item question: “How worried are you about the effect of environmental exposures on your health?” The authors also asked: “How worried are you about the effect of indoor air on your health?,” because intolerance and symptoms related to indoor air are prevalent in Finland [4]. The response options were on a scale ranging from 0 (“not at all”) to 10 (“extremely worried”) [5]. For both questions, the authors determined a cutoff level for excessive worry to distinguish high from low worries in comparison to EI.

Intolerance to chemicals is the most prevalent form of EI [4], and thus serves as its proxy. The EI is identified on the basis of self-reports by screening instruments [5,6,12]. The authors used the QEESI screening instrument, a validated questionnaire for chemical intolerance (i.e., sensitivity), and its *Chemical Intolerance* (CI) and *Symptom Severity* (SS) scales [12]. Each scale consisted of 10 items, and the participants rated the severity of their symptoms on a scale from 0 (“not a problem at all”) to 10 (“disabling symptoms”). The sum scores for each scale ranged 0–100. A sum score of ≥40 in both SS and CI was considered “very suggestive” of EI to chemicals, whereas an SS score of ≥40 and CI score of 20 to <40 was considered “somewhat suggestive” of EI to chemicals [12]. Due to the small sample numbers in our study, the authors combined the “very suggestive” and “somewhat suggestive” categories into 1 group named “individuals with EI.” The remaining respondents were categorized as “individuals without EI.” In the current study, the QEESI questionnaire was translated into Finnish, and underwent a back-translation into English before the version was finalized. Psychometric validation, such as reliability and validity assessments, was not performed for the Finnish version.

Perceived stress was assessed as: “Stress is described as a situation in which a person feels tense, restless, nervous or anxious or has difficulty sleeping because things constantly bother them. Do you currently feel this kind of stress?” The response options were: “no stress,” “somewhat stress,” “some stress,” “exceeded stress,” or “significantly exceeded stress” [17]. In statistical analyses, perceived stress was operationalized on a 5-point Likert scale ranging from 0 (“no stress”) to 4 (“significantly exceeded stress”), where higher levels indicate a less favourable condition.

Current health was evaluated by asking “In your opinion, is your current state of health compared to your age…?” with response options on a 5-point Likert scale: 0 –“excellent,” 1 – “very good,” 2 – “good,” 3 – “fair,” or 4 – “poor” [18]. In statistical analysis, higher levels of these options represent a less favourable condition.

Work ability was assessed using 2 questions from the *Work Ability Score* (WAS) [19]. The first question asked participants to rate their current work ability compared to their lifetime best on a scale from 0 (“unable to work”) to 10 (“work ability at its best”). The second question asked them to predict their work ability two years from now, with response options of “certainly,” “not sure,” or “hardly.”

The authors assessed the prevalence of 17 common diseases, such as asthma or migraine, by the asking: “Has your doctor diagnosed you with any of the following diseases?” The participants were also asked “Do you currently smoke (smoked in the past month)?” with response options of “yes” and “no.”

### Statistical analysis

The authors describe the study population using number and percent, median and quantiles. To compare the demographic characteristics of the participants and non-participants, the authors used the t-test and χ^2^ test. Dependencies between numerical and nominal variables were analysed using the Kruskal-Wallis test. The authors used the receiver operating characteristic (ROC) curve and the Youden index to determine cutoff points and to distinguish between low and high health worries about environmental exposures, both in general and in terms of indoor air, and to compare individuals with and without EI.

The authors used linear regression to illustrate the explanatory variables for health worries about environmental exposures. Model 1 is the unadjusted, i.e., crude model. In the assessment of work ability, lower levels indicate a less favourable condition, whereas for perceived stress and current health, lower levels signify a better state of condition. Model 2 includes work ability, perceived stress and self-assessed health and is adjusted for age, EI, sex, occupational group and number of diagnosed diseases.

The main variables were environmental-related health worries and were elicited using 2 questions:

– worries about the effect of environmental exposures on health,
– worries about the effect of indoor air on health.

The authors used gender, age and socioeconomic status (occupational group) as potential confounders. The analyses were performed using SPSS and the R 3.4.0 software version.

## RESULTS

The study group consisted of 355 participants, aged 17–70 years (mean (M) = 39, standard deviation (SD) = 13), with females overrepresented (54%, N = 193). Non-respondents (N = 264) were slightly younger, with an average age of 37 years (SD = 12, range 17–66 years). The response rates of the males (46%) and females (54%) differed, but the difference was not statistically significant (χ^2^ = 1.888, p = 0.1694).

Table 1 presents the demographic data. The participants (N = 355) consisted of blue-collar workers (53%, N = 188), clerical employees (27%, N = 95) and managers/professionals and entrepreneurs (16%, N = 56). The majority (87%) reported being non-smokers. The most common self-reported physician-diagnosed diseases among the participants were degenerative spine disease (19%), allergic conjunctivitis (18%), atopic skin (16%) and migraine (14%).

**Table 1:** Sample characteristics of participants without and with environmental intolerance (EI)^a^, survey of working-age employees attending occupational health services health check-ups, in 2016–2018, Seinäjoki, Finland

Variable	Participants (N = 355) [n (%)]			p
	total	without EI (N = 330)	with EI (N = 25)	
Socioeconomic				
sex				**<0.001**
male	162 (45.6)	160 (48.5)	2 (8.0)	
female	193 (54.4)	170 (51.5)	23 (92.0)	
occupational group				**<0.001**
managing director	2 (0.6)	2 (0.6)	0	
entrepreneur	5 (1.4)	5 (1.5)	0	
professional/manager	49 (13.8)	47 (14.2)	2 (8.0)	
clerical employee	95 (26.8)	89 (27.0)	6 (24.0)	
blue-collar worker	188 (53.0)	173 (52.4)	15 (60.0)	
other	16 (4.5)	14 (4.2)	2 (8.0)	
marital status				**0.019**
unmarried	82 (23.1)	80 (24.2)	2 (8.0)	
married	165 (46.5)	154 (46.7)	11 (44.0)	
partnership	85 (23.9)	76 (23.0)	9 (36.0)	
divorced	21 (5.9)	18 (5.5)	3 (12.0)	
widow	2 (0.6)	2 (0.6)	0	
Current smoking status				0.326
smoker	48 (13.5)	43 (13.0)	5 (20.0)	
non-smoker	307 (86.5)	287 (87.0)	20 (80.0)	
Medical (diseases diagnosed by physician)				
asthma	32 (9.0)	25 (7.6)	7 (28.0)	**0.004**
allergic rhinitis	23 (6.5)	63 (19.1)	5 (20.0)	>0.05
allergic conjunctivitis	63 (17.7)	21 (6.4)	2 (8.0)	>0.05
atopic skin	56 (15.8)	47 (14.2)	9 (36.0)	**0.008**
migraine	48 (13.5)	39 (11.8)	9 (36.0)	**0.001**
fibromyalgia	5 (1.4)	4 (1.2)	1 (4.0)	>0.05
chronic obstructive pulmonary disease	1 (0.3)	1 (0.3)	0	>0.05
depression	18 (5.1)	15 (4.5)	3 (12.0)	>0.05
anxiety disorder	11 (3.1)	9 (2.7)	2 (8.0)	>0.05
other mental disorder	2 (0.6)	–	2 (8.0)	**0.004**
irritable bowel syndrome	9 (2.5)	6 (1.8)	3 (12.0)	**0.016**
rheumatoid arthritis	19 (5.4)	18 (5.6)	1 (4.0)	>0.05
degenerative spine disease	66 (18.6)	62 (18.8)	4 (16.0)	>0.05
hypertension	45 (12.7)	40 (12.1)	5 (20.0)	>0.05
coronary heart disease	2 (0.6)	2 (0.6)	0	>0.05
diabetes mellitus	6 (1.7)	6 (1.8)	0	>0.05
cancer	5 (1.4)	5 (1.5)	0	>0.05
other illnesses or diseases	51 (14.4)	44 (13.3)	7 (28.0)	>0.05

Bolded are statistically significant values.

a Environmental intolerance is defined by a *Quick Environmental Exposure and Sensitivity Inventory's* (QEESI) *Symptom Severity* score of ≥40 and a *Chemical Intolerance* score of ≥20 to <40.

Of the participants, 7.0% (25 out of 355) were classified as having EI based on the chosen QEESI criteria. Significant differences were found between “individuals with EI” (N = 25) and “individuals without EI” (N = 330). The EI group members were nearly all female (N = 23 out of 25, 92%), and had higher rates of physician-diagnosed asthma, atopic skin, migraine, other mental disorders, and irritable bowel syndrome (Table 1). Those with EI reported poorer health, reduced work ability and higher perceived stress levels than those without EI (Table 2). Those with EI also expressed more health worries about both environmental exposures in general and indoor air than those without (Table 2).

**Table 2:** Associations between health measures and health worries about environmental exposures among participants with and without environmental intolerance (EI)^a^: survey of working-aged employees attending occupational health services health check-ups, in 2016–2018, Seinäjoki, Finland

Variable	Participants (N = 335)			p^b^
	total	without EI (N = 330)	with EI (N = 25)	
Current health [n (%)]				**<0.001**
excellent	44 (12.4)	43 (13.0)	1 (4.0)	
very good	163 (45.9)	156 (47.3)	7 (28.0)	
good	87 (24.5)	80 (24.2)	7 (28.0)	
fair	53 (14.9	47 (14.2)	6 (24.0)	
poor	8 (2.3)	4 (1.2)	4 (16.0)	
Self-assessed work ability (scale 0–10) [M (Q1–Q3)]	9 (8–9)	9 (8–9)	8 (6–9)	**<0.001**
Own prognosis of work ability 2 years from now [n (%)]^c^				**0.002**
certainly	332 (94.1)	312 (95.1)	20 (80.0)	
not sure	17 (4.8)	14 (4.3)	3 (12.0)	
hardly	4 (1.1)	2 (0.6)	2 (8.0)	
Perceived stress [n (%)]				**<0.001**
no stress	64 (18.0)	64 (19.4)	–	
somewhat stress	150 (42.3)	146 (44.2)	4 (16.0)	
some stress	104 (29.3)	89 (27.0)	15 (60.0)	
exceeded stress	31 (8.7)	27 (8.2)	4 (16.0)	
significantly exceeded stress	6 (1.7)	4 (1.2)	2 (8.0)	
Health worries (scale 0–10) (Me [Q1–Q3])^c^				
about environmental exposures	2 (1–4)	2 (0–3)	6 (3–8)	**<0.001**
about indoor air	3 (1–6)	2 (1–6)	7 (4–8)	**<0.001**

Q1–Q3 – first quartile – third quartile.

Bolded are statistically significant values.

a EI is defined as a *Quick Environmental Exposure and Sensitivity Inventory's* (QEESI) *Symptom Severity* score of ≥40 and a *Chemical Intolerance* score of ≥20 to <40.

b Chi-squared test/Mann-Whitney U test.

c Two answers are missing in the “without EI” group.

**Figure 2:**
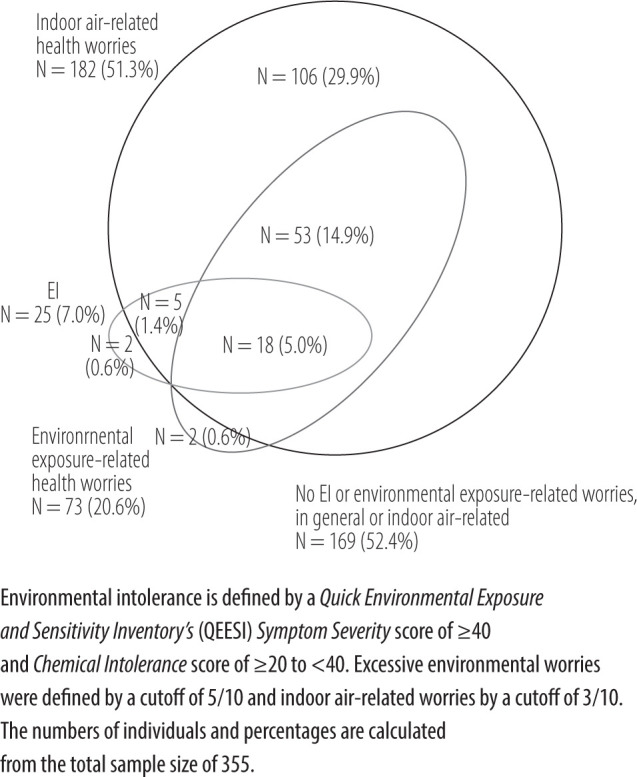
Co-occurrence of environmental intolerance (EI), excessive environmental exposure-related health worries, both general and indoor air-related, 2016–2018, Seinäjoki, Finland

The question on environmental exposure-related health worries showed a sensitivity of 0.72 and specificity of 0.83 to identifying EI, with an ROC value of 0.82 at a cutoff of 5/10. For indoor air-related health worries, sensitivity was 0.92 and specificity 0.52, with an ROC value of 0.77 at a cutoff of 3/10. Figure 2 shows the co-occurrence of EI with the 2 health worry questions on general and indoor air, using the cutoff points to indicate excessive worry. Individuals with excessive health worries about environmental exposure (21%, N = 73 out of 355) made up 75% (N = 18 out of 25) of the EI cases, and those with indoor air-related health worries (51%, N = 182 out of 355) included nearly all the EI cases (92%, N = 23 out of 25). Females were increasingly overrepresented when EI and health worries overlapped and increased: 52% (N = 56 out of 106) or 50% (N = 1 out of 2) with either 1 of the 2 health worries, 66% (N = 35 out of 53) with both health worries but no EI, 50% (N = 1 out of 2) with only EI, and nearly all (94%, N = 17 out of 18) with both EI and additional health worries (Figure 2).

Among all the participants (N = 355), linear regression analyses revealed that when the severity of their health worries about environmental exposures increased, their work ability decreased significantly (p < 0.001 in the unadjusted and p < 0.002 in the adjusted model), their perceived stress increased (p < 0.001 in both models), and their current health worsened (i.e., higher levels on the Likert scale, p < 0.001 in both models). These associations were significant in both the unadjusted and adjusted models, as shown in Table 3. Similar associations were observed for health worries about indoor air, although they did not reach statistical significance (p > 0.05) (not shown in Table 3).

**Table 3:** Linear regression analysis of health worries about environmental exposures in relation to work ability, perceived stress, and self-assessed health (N = 355): survey of working-aged employees in occupational health services health check-ups in 2016–2018, Seinäjoki, Finland

Variable	Model 1 (unadjusted)			Model 2 (adjusted)		
	β	95% CI	p	β	95% CI	p
Work ability			**<0.001**			**<0.002**
intercept	8.72	8.52–8.92		11.54	9.70–13.39	
health worries about environmental exposures	–0.12	–0.18–(–0.07)		–0.09	–0.15–(–0.04)	
environmental intolerance						
without EI (QEESI–)^a^				ref.	ref.	
with EI (QEESI+)				–0.74	–1.29–(–0.19)	
sex						
male				ref.	ref.	
female				0.36	0.07–0.64	
age				–0.02	–0.03–(–0.01)	
occupational group						
managing director				ref.	ref.	
entrepreneur				–1.75	–3.83–0.32	
professional/manager				–1.77	–3.55–0.02	
clerical employee				–2.33	–4.10–(–0.57)	
blue-collar worker				–2.17	–3.93–(–0.41)	
other				–2.48	–4.35–(–0.61)	
number of diseases				–0.01	–0.10–0.08	
Perceived stress			**<0.001**			**<0.001**
intercept	2.09	1.95–2.22		2.27	0.98–3.57	
health worries about environmental exposures	0.10	0.06–0.13		0.07	0.03–0.11	
environmental intolerance					–	
without EI (QEESI–)				ref.	ref.	
with EI (QEESI+)				0.53	0.15–0.92	
sex						
male				ref.	ref.	
female				0.27	0.07–0.47	
age				–0.01	–0.02–0.001	
occupational group						
managing director				ref.	ref.	
entrepreneur				–0.06	–1.52–1.39	
professional/manager				–0.06	–1.19–1.32	
clerical employee				–0.06	–1.29–1.18	
blue-collar worker				–0.11	–1.34–1.13	
other				–0.03	–1.34–1.28	
number of diseases				0.03	–0.04–0.09	
Current health			**<0.001**			**<0.001**
intercept	2.26	2.12–2.41		1.12	–0.24–2.48	
health worries about environmental exposures	0.09	0.05–0.12		0.08	0.03–0.12	
environmental intolerance						
without EI (QEESI–)				ref.	ref.	
with EI (QEESI+)				0.60	0.19–1.00	
sex						
male				ref.	ref.	
female				–0.29	–0.50–(–0.08)	
age				0.001	–0.01–0.01	
occupational group						
managing director				ref.	ref.	
entrepreneur				1.20	–0.33–2.72	
professional/manager				0.10	–0.32–2.31	
clerical employee				1.45	0.15–2.75	
blue-collar worker				1.20	–0.10–2.49	
other				1.24	–0.13–2.62	
number of diseases				0.002	–0.07–0.07	

β – regression coefficient, EI - environmental intolerance; QEESI – *Quick Environmental Exposure and Sensitivity Inventory*; ref. – reference.

The table presents both the unadjusted and adjusted linear regression models, with adjustments for EI, age, sex, occupational group and number of physician-diagnosed diseases. In the analysis, on the ascending scales, lower values of work ability indicate a less favourable condition, whereas lower values of perceived stress and current health indicate a more favourable condition.

Bolded are statistically significant values.

a EI is defined as a *Quick Environmental Exposure and Sensitivity Inventory's* (QEESI) *Symptom Severity* score of ≥40 and a *Chemical Intolerance* score of ≥20 to <40.

## DISCUSSION

The authors' study shows the association between health worries about environmental exposures, both general and indoor air-related, and self-reported EI, poor work ability, and negative health outcomes. Of the employees attending OH check-ups, 7% reported EI, 21% expressed excessive health worries about environmental exposures, and 51% were worried about their indoor air-related health. The single-item questions on health worries effectively identified EI, and those reporting excessive health worries about environmental exposure largely included the EI cases. The participants identified as having EI by the QEESI, a validated questionnaire on environmental intolerance to chemicals, had a higher prevalence of comorbid diseases, poorer current health, more perceived stress, and poorer work ability than those without EI. Among all the participants, the severity of health worries about environmental exposures, identified by a single-item question, was associated not only with self-reported EI but also with poorer work ability, higher stress levels, and poorer current health. The health worries about indoor air behaved similarly to those about environmental exposures in general, but their associations with poorer work ability and health remained statistically insignificant.

Although the data set was not optimal for prevalence estimation, the authors found a 7% prevalence of EI, determined by symptoms and reactions to chemicals using the QEESI. This is in line with a previous study among fertile-aged women in Finland, where 9.9% reported intolerance to chemicals with behavioural changes and 5.7% experienced intolerance-related disability [6]. These rates are comparable to those from an earlier study of the general Finnish population, in which 15% of the participants reported intolerance to chemicals in a single-question assessment [4]. Excessive health worries about environmental (21%) and indoor (51%) exposures were higher than expected, in comparison to the authors' prevalence of EI, and the 18% of adults in New Zealand who reported any health-affecting worry [13].

The QEESI criteria used, including both the “somewhat and very suggestive” of EI to chemicals, identify individuals with symptoms and reactions related to various odours or chemical exposures [12]. Thus, not surprisingly, the findings revealed a significant interaction between symptomatic EI and impaired work ability and overall health, supporting the results of previous studies [6] that have included the aspect of symptoms and reactivity to environmental factors in their EI definition.

Consistent with previous research, the participants in the authors' study with identified EI showed significantly reduced work ability [10,20], elevated stress levels [10,21], comorbidities, poorer current health [10,11]. The prevalence of these factors were higher among females [1,4,8]. According to the nature of perceived suffering in EI and other functional disorders, self-assessments of functioning (e.g., work ability) in psychosocial (activity and participation) settings show more severe disability than objective medical evaluations [5]. The authors' study also revealed that environmental-related worries were associated with high rates of somatic and psychiatric comorbidities, such as asthma, depression, and anxiety, in line with earlier clinical [5] and population-based studies [22]. Mental health comorbidities, psychological distress and catastrophizing are linked to more severe disabilities from environmental-related symptoms and illness and disease in general [14,16,23]. Depression and anxiety harbours increased health-related worries among those with bodily preoccupations than among those without these conditions [24]. A vicious cycle of concerns and catastrophizing with misattribution and nocebo labelling of the environment can exacerbate mental distress and symptoms, and *vice versa* [15].

The authors' particular focus was on health worries about environmental exposures and their association with adverse health outcomes and EI. Health worries and EI, here defined as bodily reactivity to the chemical environment, significantly overlapped with increased worries about the health effects of environmental exposures. This supports the apprehension that heightened risk perceptions and worries about the adverse health effects of environmental factors are the core contributory factors of the development and persistence of EI [1,5,15]. Worries and nocebo expectations induce central nervous system stress mechanisms, leading to a wide range of illness and health outcomes [1,15]. The authors' findings suggest that risk perceptions should be incorporated in the assessment of EI and health surveys, which can serve as targets of preventive health and interventions.

In order to identify excessive health worries, the authors configured cutoffs by their association with EI, as defined by the QEESI. Excessive health worries outnumbered and included the EI cases, suggesting a continuum from health worries to EI with bodily reactivity. Consistent with previous findings, the authors observed that females were overrepresented, with their numbers increasing as the environmental health worries increased, and nearly all the EI cases were female.

In the authors' study, health worries about environmental exposures were strongly linked to perceived stress, reduced work ability and poorer overall health in the authors' sample of working-aged employees, even after the authors adjusted for variables such as age, sex, occupational group and diagnosed diseases. This is in line with previous studies, which show that risk perceptions and symptom awareness can independently influence health outcomes and contribute to a cycle of negative consequences [8–10,16]. Perceived environmental health risks can lead to increased symptom reporting [25]. These results highlight the need to include and shift the focus from exposure and reactivity to risk perception [2].

The authors found that health worries about indoor air, which are very common in Finland, were particularly prevalent and overlapped with EI and worries about environmental exposures in general. This reflects the regional cultural and societal risk perceptions that affect symptom attribution, which is supported by the finding that building-related EI is more common in Finland than in Sweden [4]. Also, parental worries about indoor environmental quality are associated with more symptom reports by children [26]. This highlights the psychosocial transmission of perceived health risks and reactivity [27]. Although health worries about indoor air included nearly all cases of EI and environmental-related worry in general, they did not show a significant association with adverse health outcomes. This may represent a milder form of health worries than general environmental worries and EI, and include a larger proportion of individuals at the milder end of the continuum with less symptom misattribution, while also reflecting the general population's risk perceptions. Maladaptive manifestations of illness behaviour, such as health anxiety and catastrophizing, can increase and affect symptoms and reactivity, and hinder the prevention and treatment of medical conditions [28]. Moreover, chronic stress, or allostatic load, is a critical psychosocial factor that affects individual vulnerability and the course of medical conditions and their outcomes. Unfortunately, these factors are often overlooked in medical settings [28]. Adopting a biopsychosocial approach enables comprehensive assessment of patient beliefs and worries, satisfactory patient-doctor interactions, and personalized care [28]. Although further prospective studies are needed, our results support the importance of evaluating illness behaviour, particularly excess health worries and their attributions (e.g., to environmental exposures), and their association with adverse health outcomes and disability. In clinical practice, a single question on health worries about environmental exposures seems to serve early identification. Addressing these worries and their impact on functioning can lead to treatment of EI [10] and preventive actions, rather than focusing on symptoms, exposures and medical and toxicological issues.

The uniqueness and the strength of this study is that the survey was conducted during preventive OH check-ups. As the selected employees had not been referred to OH due to specific symptoms, illnesses or work-related adverse effects, the sample is representative of working-aged employees in general. This OH setting minimizes Berkson's bias, often present in studies targeting populations seeking medical care, and may provide a less biased group. Although the participation rate was low at 36%, it was sufficient for the study's purpose and enabled significant results. Also, the authors' questions on EI and health worries were part of a broader health questionnaire, which reduced the likelihood of attracting only those who were excessively worried about environmental issues.

The study also has limitations. The first is that the data were self-reported. the authors' 2 single-item questions on environmental health worries had not been validated in epidemiological studies, although they had proven useful in a clinical study of patients with indoor air-related EI [5]. Another is that the cohort was rather small, which resulted in a limited sample size of individuals with EI (N = 25 out of 355). Also, the cross-sectional design precludes the assessment of causality, which may have led to selection bias. Finally, individuals with more severe symptoms may be absent from work, and so the participants may have been the healthiest employees, resulting in the healthy worker effect. Therefore, more widely generalizing the prevalence data requires caution.

Future research should focus on psychosocial risk perceptions ranging from worry to catastrophizing, as these can significantly influence the initiation and persistence of symptoms and disability in individuals. Given that repeated symptom queries may lead to increased symptom reporting [29], the assessment of risk perceptions related to the environment could provide grounds for a more biopsychosocial approach. Further studies are required to validate and test the usability of these one-item questions and to develop preventive measures for risk-evoked disabilities as well as the Finnish version of the QEESI questionnaire.

## CONCLUSIONS

Single-item questions can identify environmental exposure-related health worries and EI that are associated with reduced work ability and ill health. These seem to be on the same continuum of increasing risk perception of environmental exposures. Using a biopsychosocial approach to assessing excess risk perceptions enables early detection and fosters preventive interventions, such as psychoeducation.
